# WNK3 Maintains the GABAergic Inhibitory Tone, Synaptic Excitation and Neuronal Excitability *via* Regulation of KCC2 Cotransporter in Mature Neurons

**DOI:** 10.3389/fnmol.2021.762142

**Published:** 2021-11-10

**Authors:** Wee Meng Lim, Eunice W. M. Chin, Bor Luen Tang, Tingting Chen, Eyleen L. K. Goh

**Affiliations:** ^1^Neuroscience Academic Clinical Programme, Duke-NUS Medical School, Singapore, Singapore; ^2^NUS Graduate School for Integrative Sciences and Engineering, Singapore, Singapore; ^3^Neuroscience and Mental Health Faculty, Lee Kong Chian School of Medicine, Nanyang Technological University, Singapore, Singapore; ^4^Department of Biochemistry, Yong Loo Lin School of Medicine, National University of Singapore, Singapore, Singapore; ^5^School of Pharmacy, Nantong University, Nantong, China

**Keywords:** WNK3, GABAergic inhibitory tone, synaptic excitation, hyperpolarized *E*_**GABA**_, neuronal excitability, KCC2 cotransporter

## Abstract

The activation of chloride (Cl^−^)permeable gamma (γ)-aminobutyric acid type A(GABA_A_) receptors induces synaptic inhibition in mature and excitation in immature neurons. This developmental “switch” in GABA function controlled by its polarity depends on the postnatal decrease in intraneuronal Cl^−^ concentration mediated by KCC2, a member of cation-chloride cotransporters (CCCs). The serine-threonine kinase WNK3 (With No Lysine [K]), is a potent regulator of all CCCs and is expressed in neurons. Here, we characterized the functions of WNK3 and its role in GABAergic signaling in cultured embryonic day 18 (E18) hippocampal neurons. We observed a decrease in WNK3 expression as neurons mature. Knocking down of WNK3 significantly hyperpolarized *E_GABA_* in mature neurons (DIV13–15) but had no effect on immature neurons (DIV6–8). This hyperpolarized *E_GABA_* in WNK3-deficient neurons was not due to the total expression of NKCC1 and KCC2, that remained unchanged. However, there was a reduction in phosphorylated KCC2 at the membrane, suggesting an increase in KCC2 chloride export activity. Furthermore, hyperpolarized *E_GABA_* observed in WNK3-deficient neurons can be reversed by the KCC2 inhibitor, VU024055, thus indicating that WNK3 acts through KCC2 to influence *E_GABA_*. Notably, WNK3 knockdown resulted in morphological changes in mature but not immature neurons. Electrophysiological characterization of WNK3-deficient mature neurons revealed reduced capacitances but increased intrinsic excitability and synaptic excitation. Hence, our study demonstrates that WNK3 maintains the “adult” GABAergic inhibitory tone in neurons and plays a role in the morphological development of neurons and excitability.

## Introduction

GABA is a major inhibitory neurotransmitter of the mature central nervous system (CNS) that plays a crucial role in controlling neuronal excitability in the brain (Ben-Ari et al., 2012). The direction of GABA currents through ionotropic GABA receptors reverses from depolarizing to hyperpolarizing as the brain develops (Ben-Ari, 2014; Peerboom and Wierenga, 2021). This developmental “switch” in GABA function is controlled by its polarity of transmission, which can be reflected by the equilibrium potential of GABA (*E_GABA_*; Ouardouz and Sastry, 2005). The amplitude and direction of GABA_A_Rs-mediated postsynaptic currents are subjected to *E_GABA_*, and the shifts of *E_GABA_* can affect GABAergic neurotransmission, neuron excitability, and excitatory synaptic plasticity (Ben-Ari, 2014; Peerboom and Wierenga, 2021).

GABA_A_Rs are predominantly ligand-gated Cl^−^ channels. *E_GABA_*, which reflects the strength and polarity of GABAergic neurotransmission, is determined by the concentration of intracellular Cl^−^([Cl^−^]i) in neurons (Farrant and Kaila, 2007). In the developing brain, [Cl^−^]i is relatively high resulting in a depolarization effect of *E_GABA_* (Ben-Ari et al., 2012). The depolarizing activity of GABAergic signaling is critical for neuronal proliferation and migration, and synaptogenesis (Ben-Ari et al., 2007; Wang and Kriegstein, 2009). Conversely, in the adult brain, [Cl^−^]i is maintained at low levels, thus resulting in a hyperpolarizing effect of GABAergic signaling. In this way, the *E_GABA_* in neonatal neurons is set at a relatively depolarized level, but subsequently shows a negative shift in the adult brain neurons (Yang et al., 2010). This change in GABA polarizing state is due to the cation chloride cotransporters (CCCs; Rivera et al., 1999; Ouardouz and Sastry, 2005). However, despite the local depolarization effect, immature GABA_A_ transmission has inhibitory effects *in vivo* (Kirmse et al., 2015; Oh et al., 2016; Valeeva et al., 2016). This ability of GABA to be simultaneously depolarizing and inhibitory can be due to a shunting inhibition action resulting from the opening of GABA_A_Rs. Decreases in input resistance and membrane time constant when GABA_A_ receptors open, act by decreasing the temporal and spatial summation of excitatory inputs, regardless of the direction of Cl^−^ flux (Blaesse et al., 2009).

CCCs play a crucial role in regulating chloride gradients that control the [Cl^−^]i in neurons. Two members of CCCs, Na^+^ -K^+^-Cl^−^ cotransporter 1 (NKCC1) and K^+^-Cl^−^ cotransporter 2 (KCC2), play prominent roles in affecting intracellular chloride concentration as key neuronal Cl^−^ importer and exporter, respectively. NKCC1 and KCC2 belong to a family of secondary active cotransporters, which are involved in intracellular Cl^−^ regulation and cell volume regulation (Blaesse et al., 2009), and their activities are regulated through surface expression and phosphorylation (Come et al., 2020). Cl^−^ homeostasis depends on developmental changes in NKCC1 and KCC2 expression (Watanabe and Fukuda, 2015). Due to difficulties in NKCC1 detection in adult animals, the literature on the developmental pattern of NKCC1 mRNA and protein expression are highly contradictory (Virtanen et al., 2020). In the human and rodent forebrain, several studies have reported a robust developmental upregulation (Hyde et al., 2011; Morita et al., 2014), while several others observed a clear downregulation (Yamada et al., 2004; Lee H. A. et al., 2010). In contrast, KCC2 is almost exclusively expressed in the CNS and is upregulated as neurons mature (Rivera et al., 1999; Acton et al., 2012). Postnatal upregulation of KCC2 expression has been shown to contribute to a progressive shift from depolarizing of GABAergic transmission in the CNS during early development to hyperpolarizing during adulthood (Rivera et al., 1999; Ludwig et al., 2003; Deidda et al., 2014). In addition to its function in GABA neurotransmission, KCC2 is also critical for dendritic spine formation (Li et al., 2007; Fiumelli et al., 2013; Awad et al., 2018) and the function of excitatory synapses (Gauvain et al., 2011). A direct association between KCC2 dysfunction and numerous psychiatric or neurological disorders (Kahle et al., 2008; Kaila et al., 2014), including epilepsy (Di Cristo et al., 2018), Huntington’s disease (Dargaei et al., 2018), schizophrenia (Hyde et al., 2011), and Rett syndrome (Tang et al., 2016), has been reported.

With No Lysine[K] (WNK) is a serine-threonine kinase that has four known subtypes (WNK1, WNK2, WNK3, and WNK4) expressed in the mammalian brain (Wilson et al., 2001; Kahle et al., 2006). Both KCC and NKCC are regulated by WNKs, *via* phosphorylation to inhibit or enhance their activities respectively (Kahle et al., 2006, 2010). WNK1 is expressed in most tissues examined (Zambrowicz et al., 2003), and has recently been shown to regulate GABAergic response through KCC2 phosphorylation in immature neurons (Friedel et al., 2015). WNK2 is predominantly expressed in the heart, brain, and colon, and may act as a tumor suppressor in neurons (Rinehart et al., 2011). WNK4 is mainly found in the colon and skin (Zambrowicz et al., 2003), while its expression in the brain is restricted to endothelial cells that form the blood-brain barrier (Kahle et al., 2004). Verissimo and Jordan (2001) have conducted RT-PCR analysis for the expression of *WNK1*, *WNK2*, *WNK3*, and *WNK4* in fetal and adult human tissues. Notably, *WNK3* is brain-enriched (Verissimo and Jordan, 2001). Specifically, the *WNK3* splice isoform containing exon 18b was found to be restricted to the fetal and adult brain and was detected in areas of the adult brain required for memory, cognition, and movement (Holden et al., 2004). *Wnk3* is highly expressed in the fetal brain and its levels has been reported to increase during early postnatal development [absent at postnatal day 10 (P10), becoming highly expressed at P21; Kahle et al., 2005]. However, changes in WNK3’s protein expression during neuronal maturation and its role in development have not been clearly demonstrated. WNK3 exerts a stronger inhibitory effect than WNK2 on the activity of all four KCCs isoforms (Rinehart et al., 2011). In particular, the spatial and temporal expression pattern of WNK3 closely parallels that of KCC2 (Lu et al., 1999). An increase in expression and activity of WNK3 was thought to be the critical cause of GABA-mediated neurotransmission disturbance in CNS, consequently leading to schizophrenia (Spitzer, 2010; Arion and Lewis, 2011). Interestingly, WNK3 knockout has a neuroprotective effect to mice in ischemia model (Begum et al., 2015).

In this study, we have examined the functional role of WNK3 through *E_GABA_* regulation, morphological, neuronal excitatory, and synaptic excitation changes, using lentiviral-mediated knockdown of WNK3 in cultured hippocampal neurons as an *in vitro* neuronal maturation model. We found that WNK3 knockdown induced a hyperpolarizing shift of *E_GABA_* through the activation of KCC2 in functionally mature neurons, but not in immature neurons. Mature WNK3 knockdown neurons also showed alteration in morphology, neuron intrinsic excitability, and synaptic excitation. Our data suggest that WNK3 plays an important role in the proper functioning of adult cultured hippocampal neurons.

## Materials and Methods

### Cloning of Lentiviral Constructs and Virus Production

FUGW lentiviral constructs (Addgene) were modified to carry both a GFP reporter gene driven by human ubiquitin C promoter, and a control short hairpin RNA (shRNA) driven by U6 promoter. The schematic diagram for both constructs is illustrated in Figure 2A. shRNA sense sequences were designed to target rat WNK3 (NM_001163607.1, *Wnk3*sh1—GGGTTGAAGATCCTAAGAA, *Wnk*3sh2—GGGACTAAATTCCAGCTTACT). Non-specific scrambled shRNA sequence (TTATCAGATAGACGATTGT) was used as control shRNA. PCR using a common forward primer (TTAATTAAGCAGATCTGGGCAGGAAGAGGGGCTAT) and shRNA-containing oligonucleotides was cloned into the FUGW plasmid (Chew et al., 2015). Virus particles were produced by using calcium-phosphate transfection of FUGW plasmid and packaging vectors into the human embryonic kidney (HEK) 293 gp cells. Virus-containing culture media (DMEM, Invitrogen, USA) was collected and the virus concentrated *via* ultracentrifugation (25,000 *g*, 90 min). The viral pellet was then re-suspended in PBS overnight, aliquoted, and stored at −80°C until use.

**Figure 1:**
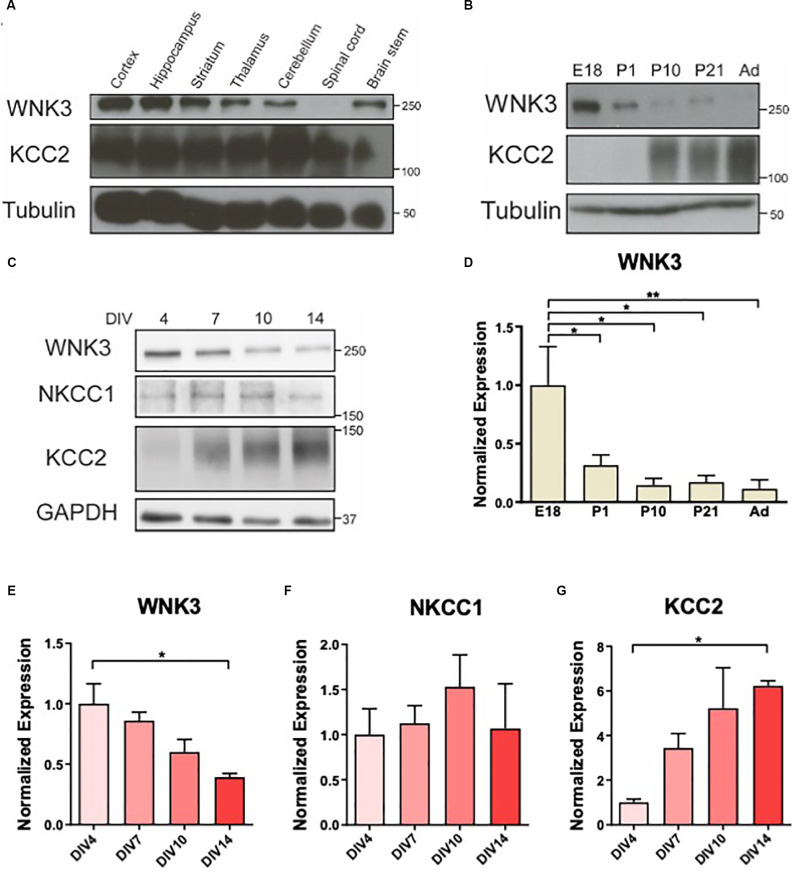
Downregulation ofWNK3 expression during maturation of cultured neurons. **(A)**Representative Western blots showing the expression of WNK3 andKCC2 in different CNS regions. **(B)** Representative Westernblots showing the expression of WNK3 and KCC2 during the developmentof hippocampus. **(C)** Representative Western blots showing theexpression of WNK3, NKCC1, and KCC2 in hippocampal neurons atdifferent stages of culture. **(D)** Quantification ofWNK3 protein expression from **(B)**, protein expression levels werenormalized to their respective loading controls and expressedrelative to E18 levels. *n* = 3 animals.*p < 0.05 and **p < 0.01. One-way ANOVAwith Bonferroni *post-hoc*. **(E–G)** Quantification ofWNK3, NKCC1, and KCC2 protein expression from **(C)**. Proteinexpression levels were normalized to their respective loadingcontrols and expressed relative to DIV4 levels.*n* = 3 cultures. *p < 0.05 and**p < 0.01. One-way ANOVA with Bonferroni*post-hoc*.

**Figure 2:**
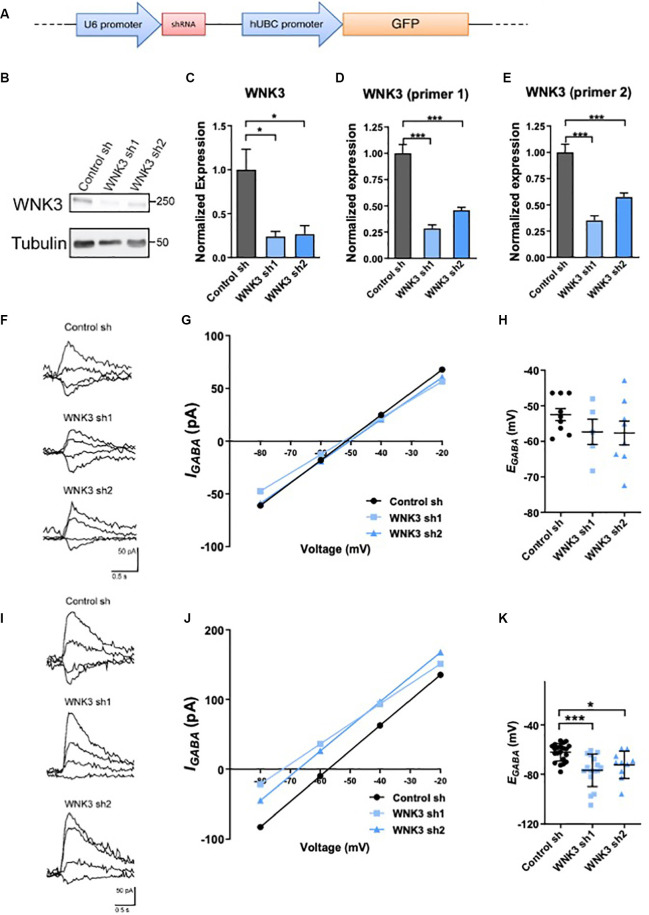
Regulation ofWNK3 on *E_GABA_* in immature and developed neurons. **(A)** The schematic diagram for FUGW lentiviral constructs carrying both a GFP reporter gene driven by human ubiquitin-C promoter, and a control short hairpin RNA (shRNA) driven by U6 promoter. **(B)** Representative Western blots showing the expression of WNK3 in WNK3 sh1 and sh2 neurons at DIV7. **(C)** Quantification of WNK3 protein expression from **(B)**. **(D,E)** Relative expression of WNK3 primer1 and primer2 mRNA in WNK3 sh1 and sh2 neurons at DIV7, protein, and mRNA expression levels were normalized to their respective loading controls and expressed relative to control sh levels.*n* = 3 cultures. *p < 0.05 and ***p < 0.001. One-way ANOVA with Bonferroni *post-hoc*. **(F)** Relative image of GABA-evoked current of control, WNK3 sh1, and sh2 neurons at DIV6–8. **(G)** Representative currents recorded in gramicidin perforated-patch configuration during incremental voltage steps and corresponding current-potential curves in control and WNK3 sh neurons at DIV6–8. *E_GABA_* is interpolated from the four points around reversal. **(H)** Summary data (control sh:*n* = 9 neurons; shwnk3 sh1: *n* = 5 neurons;WNK3 sh2: *n* = 8 neurons) of *E_GABA_*.**(I)** Relative image of GABA-evoked current of controland WNK3 sh neurons at DIV14. **(J)** Representative currents recorded in gramicidin perforated-patch configuration during incremental voltage steps and corresponding current-potential curves in control and WNK3 sh neurons at DIV14, *E_GABA_* is interpolated from the four points around a reversal. **(K)** Summary data (control sh: *n* = 9 cells; shwnk3 sh1: *n* = 5 neurons; WNK3 sh2: *n* = 8 neurons) of *E_GABA_*. *p < 0.05 and ***p < 0.001 compared to control sh. One-way ANOVA with Bonferroni *post-hoc*.

### Dissociation and Maintenance of Hippocampal Neuronal Cultures

The protocol for rat hippocampal neuronal culture has been previously established (Su et al., 2015). Briefly, Sprague-Dawley dams pregnant with E18 embryos were sacrificed in accordance with approved protocols. The brains were removed from embryos and placed in ice-cold dissection buffer (HBSS, Gibco, USA). The hippocampus and cortex were dissected from the brain and dissociated with papain (Worthington Biochemical, USA) before plating. These E18 hippocampal neurons were used for all experiments except brain region expression studies for Figure 1A. For biochemical analysis, neurons were plated at a density of approximately 15,000 cells/cm^2^ onto a 6-well plate (Corning, USA). For electrophysiological recordings, neurons were plated at a density of approximately 30,000 cells/cm^2^ per 24-well plate (Corning) onto acid-treated coverslips. The plating media (Minimum Essential Medium containing 10% fetal bovine serum, 1× N2 supplement, 3.6 mg/ml glucose, and 1× penicillin/streptomycin) was replaced by maintenance media (Neurobasal media supplemented with 1× penicillin-streptomycin, 1× B27 supplement, and 0.5× L-glutamine) the next day, and lentivirus was added a day after plating (DIV1).

### Protein Extraction and Western Blot Analysis

Whole-cell lysates or homogenized tissue lysates were extracted using a protein extraction buffer containing: 150 mM NaCl, 10 mM Tris, 1 mM EGTA, 1 mM EDTA, 1% Triton-X100 and 0.5% NP40. Cultured neurons were washed with chilled PBS before the addition of chilled protein extraction buffer. Cells were harvested by scraping in protein extraction buffer, incubated on ice for 30 min, and vortexed every 10 min. For protein extraction from the adult mice (2 months), tissues from different brain regions were dissected and immediately homogenized in protein extraction buffer with a hand-held homogenizer. Homogenized tissue lysates were also periodically vortexed (every 10 min) while incubated on ice for 30 min. Both cell and tissue lysates were cleared of debris by centrifugation (14,000 rpm, 15 min, 4°C) and measured for protein concentration before storage at −20°C until use. For membrane fractionation of neuronal cultures, cells were washed with ice-cold PBS twice before scraping in a buffer containing 50 mM Tris-HCl (pH 7.6), collected, and homogenized using a 22G syringe. Lysates were spun at 20,000 *g* for 20 min at 4°C and the supernatant was collected as the cytoplasmic fraction. The pellet was washed with ice-cold deionized water to lyse vesicles and spun again at the same setting, washed, spun for the last time before resuspension in the same buffer as the membrane fraction.

Lysates were denatured by adding SDS loading buffer and heating to 95°C for 5 min on a dry heat block. Equal protein mass for each sample were resolved on 6–10% sodium dodecyl sulfate polyacrylamide gel electrophoresis (SDS-PAGE) gels, together with protein standards (Bio-Rad, USA). The proteins were then transferred (100 V, 1.5 h) onto methanol-activated PVDF membrane (Bio-Rad) before blocking with 5% low fat dry milk in Tris- buffered saline-Tween (TBS-Tw, 0.6% Tris, 0.9% NaCl, 0.01% Tween-20) for 1 h. Primary antibodies were diluted in blocking buffer and incubated with the blots overnight at 4°C under gentle agitation. The membrane was washed with TBS-Tw thoroughly before a 1 h incubation of horseradish peroxidase (HRP)-conjugated antibody (mouse IgG HRP or rabbit IgG, GE Healthcare, USA), and washed thrice in TBS-Tw again before chemiluminescence detection.

For chemiluminescence detection, blots were incubated with Enhanced Chemiluminescence (ECL) reagent (Invitrogen), and the exposure was captured using the Image Quant LAS4000 system (GE Healthcare). The densitometry of the images was quantified using ImageJ (NIH). Briefly, rectangular boxes were drawn around each band and the densitometry was analyzed using ImageJ’s “Plot Lanes” function. Additional precaution was taken if bands are close to each other by careful visual inspection. Antibodies used in this study were: rabbit polyclonal anti-WNK3 (Millipore, USA), sheep polyclonal anti-NKCC1 (University of Dundee, USA), sheep polyclonal anti-phospho-T203/T207/T212 (pT203/pT207/pT212) NKCC1 (University of Dundee), rabbit polyclonal anti-KCC2 (07-432, Millipore), sheep polyclonal anti-pT1006 KCC2A (University of Dundee; Friedel et al., 2015), rabbit monoclonal GAPDH (Cell Signaling, USA) and rabbit polyclonal anti- Na^+^-K^+^-ATPase (Cell Signaling). There is currently no evidence indicating that Na^+^-K^+^-ATPase abundance in membrane fractions was affected by WNK.

### RNA Extraction and qPCR Analysis

Total RNA from cultured neurons was extracted at the desired time point using TRIzol™ (Invitrogen) following the manufacturer’s instructions. Complementary DNA (cDNA) was produced by reverse transcription and analyzed using a quantitative PCR (qPCR) detection system (iQ, Bio-Rad). The qPCR primers used in this study were: *Wnk3* qPCR FP1—TTCCAGCTTACTGTCCTTCAGGTCT, *Wnk3* qPCR RP1—AGTCAGCGATATCCTCAGGTGC, *Wnk3* qPCR FP2—TGCAACCTTAATGCGCACATC, *Wnk3* qPCR RP2—ACTCCGAAGTAGCCATTTCCAACA, TATA-binding protein (*Tbp*) FP—ACCGTGAATCTTGGCTGTAAAC, *Tbp* RP—CGCAGTTGTTCGTGGCTCTC, peptidyl-propyl-isomerase A (*Ppia*) FP—GTCAACCCCACCGTGTTCTTC, *Ppia* RP—ATCCTTTCTCCCCAGTGCTCAG. *Tbp* and *Ppia* were used as endogenous controls for normalizing gene expression. The results were analyzed using manufacture’s software (CFX Manager 3.1, Bio-Rad) and normalized gene expression level is calculated as 2^−ΔΔCt^, where ΔΔCt = (Ct_WNK3_*sample* − Ct_ref_*sample*) − (Ct_WNK3_*control* − Ct_ref_*control*).

### Morphological Analysis

For analysis of neuronal morphology *in vitro*, cells were imaged on a single plane at 20x magnification in the Map2 (marker for dendrites) immunofluorescence channel. Total dendritic length and number of branches were traced from images taken with a Zeiss LSM 710 confocal microscope. Images were analyzed using ImageJ and LSM Image Browser. At least 40 cells per condition from three batches of cultures were analyzed. The n-number for each cell group/condition is indicated in the results section and figure legend. Sholl analysis was performed with images of traced dendritic arbors using an ImageJ plugin (Chin et al., 2019). The sampling step size was set at 10 μm.

### Whole Cell and Gramicidin-Perforated Electrophysiological Procedures

The electrophysiological setup comprised of an inverted microscope (Axioscope A1, Carl Zeiss, Germany), a digitizer (Digidata 1440a, Molecular Devices, USA), an amplifier (Multiclamp 800b, Molecular Devices), a micromanipulator (MPC-100, Sutter Instrument, Germany), a pressure handling system (Picospritzer III, Parker Hannifin) and an excitation light source (X-Cite 120Q, Excelitas Technologies). Recordings were acquired at 10 kHz. Patch electrodes were fashioned from filamented borosilicate glass (G150F, Warner Instruments) using a vertical pipette puller (PC-10, Narashige), and range from 3 to 8 MΩ in resistance. Whole-cell recording was performed using potassium gluconate internal solution (pH 7.4) containing (in mM): 120 K-gluconate, 9 KCl, 10 KOH, 3.5 MgCl_2_, 4 NaCl, 10 HEPES, 4 Na_2_ATP, 0.4 Na_3_GTP, 0.5 EGTA, 17.5 sucrose. The external solution (pH 7.4) contained (in mM): 125 NaCl, 23 NaHCO_3_, 2.5 KCl, 0.8 NaH_2_PO_4_, 2.5 CaCl_2_, 1 MgCl_2_, 10 glucose, 0.001 tetrodotoxin (TTX), and 0.02 bicuculline methiodide (BMI). TTX and BMI were acquired from Tocris Bioscience. For gramicidin perforated patch clamp, gramicidin D (G5002, Sigma) was dissolved in DMSO at 40 mg/ml and stored at −20°C. The internal solution (pH 7.4) contained (in mM): 140 NaCl, 2.5 KCl, 2 CaCl_2_, 2 MgCl_2_, 20 HEPES, 20 glucose. The external solution (pH 7.4) contained (in mM): 150 KCl, 10 HEPES. The osmolarity of all internal and external solutions ranged around 290 and 310 mOsm/kg, respectively.

Action potentials were recorded in the current clamp and were induced by a stepwise increment of 20 pA, starting from −20 pA to 160 pA, to generate the input-output curves by plotting firing frequency in response to a series of current steps. As for gramicidin recordings, fresh gramicidin internal solutions (40 μg/ml) were prepared every 2 h. After the formation of the giga-ohm patch, the development of the perforation was monitored with continuous test pulses (−5 mV, 50 Hz, holding potential of −60 mV) and stable perforation typically took 10–15 min with series resistance typically between 40 and 60 MΩ (53.18 ± 4.565 MΩ for DIV7 neurons, *n* = 26; 46.98 ± 4.624 MΩ for DIV14 neurons, *n* = 27). The puff pipette containing 30 μM GABA dissolved in external solution was positioned within approximately 100 μm away for a test puff. The test puff is performed at −80 mV to ensure GABA-evoked peak current (*I_GABA_*) does not exceed 200 pA. This method was adapted from Friedel et al. (2015) and ensures the linearity and reproducibility of *I_GABA_* measured. Cultured hippocampal neurons were held at −60 mV before focal application of GABA (10 psi, 100 ms) while clamped at voltages between −80 mV and −20 mV, in 20 mV increments. *I_GABA_* analysis was performed using Clampfit software and *E_GABA_* was recorded as the x-intercept of the linearly fitted *I_GABA_* values for each neuron. Voltage corrections for liquid junction potential were performed offline. Recordings in which seal current deteriorates beyond −150 pA were discarded. *E_GABA_* measurements before and after bath application of NKCC1 (10 μM bumetanide, Sigma, USA) or KCC2 (80 μM VU0240551, Tocris, UK) antagonist were performed 5 min after the drug was applied and 5 min after washout.

### Data and Statistical Analysis

All statistical analyses were performed with GraphPad Prism 8. Data are presented as mean ± s.e.m unless stated otherwise. Differences among means were analyzed using analyses of variance (ANOVA) with or without repeated measures, followed by Bonferroni* post hoc* analysis. *p*- and F-values of ANOVAs are given in the results section. Differences at *p* < 0.05 were considered statistically significant. Repeats for experiments and statistical tests carried out are indicated in the figure legends and in the main text, respectively.

## Results

### WNK3 Expression Is Downregulated During Maturation of Cultured E18 Hippocampal Neurons

The developmental changes in NKCC1 and KCC2 expressions (Lu et al., 1999) resulted in a shift in GABA polarity. WNKs’ role as key regulators of these CCCs (Alessi et al., 2014) suggests that their expression may also be developmentally regulated. WNK3 and KCC2 are expressed in most CNS regions albeit at varying levels in the adult mice (2 months), except that WNK3 is absent in the spinal cord (Figure 1A). WNK3 expression in the developing hippocampus is sharply downregulated after birth (*F*_(4, 10)_= 5.388, *p* = 0.0141; Figures 1B,D), in contrast to an upregulation of KCC2. Next, we used cultured hippocampal neurons to determine if the developmental expression of WNK3 in brain tissues can be recapitulated in these neurons *in vitro* (Figure 1C). We observed declining WNK3 expression over time in cultured E18 hippocampal neurons, similar to the reduced WNK3 expression in tissue lysates from adult mice (*F*_(3, 8)_ = 6.614, *p* = 0.0147; Figures 1C,E). In contrast, KCC2 expression increased as these cultured neurons matured (*F*_(3, 8)_= 5.551, *p* = 0.0235; Figure 1G). However, no significant change was observed in NKCC1 levels (*F*_(3, 8)_= 0.4650, *p* = 0.7146; Figure 1F). Thus, our data demonstrate that WNK3 protein expression is developmentally downregulated, accompanied by an upregulation of KCC2 expression.

### WNK3 Regulates* E_GABA_* in Mature, but Not Immature Hippocampal Neurons

Although there is increasing focus on the critical role of WNK3 in affecting GABA-mediated neurotransmission, WNK3’s potential role in regulating* E_GABA_* in a neuronal setting has not been demonstrated. Since expression of WNK3 is developmentally regulated, we further tested if its expression change will induce a developmental shift in the polarity of GABA neurotransmission. Lentiviral shRNAs were used to knockdown WNK3 (shWNK3; Figures 2A–E). WNK3 protein levels were significantly reduced in shWNK3-expressing hippocampal neurons when compared to control neurons at DIV7 (control sh vs. WNK3 sh1: *p* = 0.0336, *n* = 3 batches of cultures, control sh vs. WNK3 sh2: *p* = 0.0434, *n* = 3 batches of cultures; Figures 2B,C). *Wnk3* mRNA levels detected using WNK3 primer 1 set (control sh vs. WNK3 sh1: *P* = 0.0002, *n* = 3 batches of cultures, control sh vs. WNK3 sh2: *p* = 0.0007, *n* = 3 batches of cultures; Figure 2D) and primer 2 set (control sh vs. WNK3 sh1: *p* = 0.0004, *n* = 3 batches of cultures, control sh vs. WNK3 sh2: *p* = 0.0008, *n* = 3 batches of cultures; Figure 2E) also demonstrated effective knockdown. We next examined if WNK3 regulates GABAergic transmission in immature (DIV6–8) and mature (DIV13–15) hippocampal neurons, by using gramicidin-perforated patch clamp and measuring *E_GABA_* (Figures 2F–K). WNK3 knockdown did not significantly alter *E_GABA_* in immature neurons (DIV6–8; *F*_(2, 19)_= 1.191, *p* = 0.3257; control sh: −52.47 ± mV, WNK3 sh1: −57.36 ± mV, *p* = 0.18, *n* = 9, 5 neurons; control sh vs. WNK3 sh2: −57.66 ± mV, *p* = 0.174, *n* = 9, 8 neurons; Figures 2F–H). However, *E_GABA_* was at −62.25 ± 7.086 mV in mature (DIV 14) control neurons, and −76.84 ± 13.04 mV and −72.39 ± 11.03 mV in mature WNK3 sh1 and sh2 neurons respectively, with a −14 mV and −10 mV hyperpolarizing shift of *E_GABA_*respectively (*F*_(2, 44)_= 9.684, *p* = 0.0003; control vs. WNK3 sh1: *p* = 0.0002, *n* = 21, 16 neurons; control vs. WNK3 sh2: *p* = 0.0264, *n* = 21, 10 neurons; Figures 2I–K). These results suggest that in mature neurons but not immature neurons, WNK3 knockdown induced a hyperpolarizing shift of GABA, likely resulting from a reduction of intracellular Cl^−^ concentration under the stronger negative driving force of Cl^−^.

### *E_GABA_* Hyperpolarization in Mature shWNK3 Hippocampal Neurons Is due to Overactive KCC2 Chloride Transport

**Figure 3:**
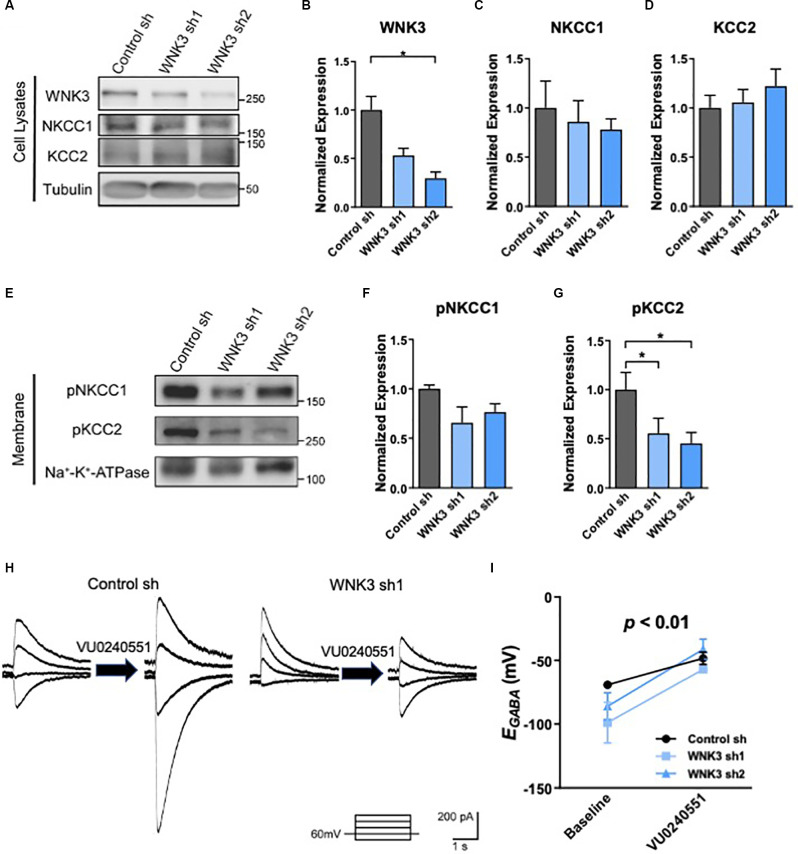
The role ofKCC2 chloride transport in *E_GABA_*hyperpolarization in shWNK3 developed neurons. **(A)**Representative Western blots showing the expression ofWNK3, NKCC1, and KCC2 in WNK3 sh1 and sh2 neurons at DIV 14.**(B–D)** Quantification of WNK3, NKCC1, andKCC2 protein expression from **(A)**, protein expression levelswere normalized to their respective loading controls and expressedrelative to control sh levels, *n* = 3 cultures,*p < 0.05, One-way ANOVA with Bonferroni *post-hoc*. **(E)**Representative Western blots showing the membrane expression of phosphorylated NKCC1 and KCC2 in WNK3 sh1 and sh2 neurons at DIV 14. **(F,G)** Quantification of phosphorylated NKCC1 and KCC2 protein expression in membrane from **(E)**, protein expression levels were normalized to Na*-K^+^-ATPase, and expressed relative to control sh levels, *n* = 3 cultures, *p < 0.05, One-way ANOVA with Bonferroni *post-hoc*. **(H)** Representative image of GABA-evoked current under the effect of selective KCC2 inhibitor VU0240551 of control sh and WNK3 sh1 neurons at DIV14. **(I)** Summary data (control sh: *n* = 5 neurons; shwnk3 sh1: *n* = 3 neurons; WNK3 sh2: *n* = 5 neurons) of *E_GABA_* at baseline and with VU0240551.

WNKs have been shown to directly phosphorylate chloride channel activity-regulating kinases SPAK and OSR1, which in turn phosphorylate KCC2 at Thr1007 (Kenter et al., 1989; Rinehart et al., 2009; de Los Heros et al., 2014). Both SPAK and OSR1 kinases also play a role in modulating NKCC1 activity (Vitari et al., 2006). WNKs were reported to interact with a yet unknown kinase to phosphorylate KCC2 at Thr906 (de Los Heros et al., 2014; Friedel et al., 2015). Phosphorylation of NKCC1 and KCC2 alters their transporter activities, as well as neuronal [Cl-]i and GABAergic signaling (Kahle et al., 2005, 2013; Rinehart et al., 2009; de Los Heros et al., 2014). Thus, we next examined the phosphorylation states of NKCC1 and KCC2 in WNK3-deficient hippocampal neurons. Since NKCC1 and KCC2 are membrane proteins, we examined for phosphorylation of these proteins in the membrane fraction and cell lysates for comparisons. As compared to control neurons, WNK3 is downregulated in the cell lysates in the WNK3 sh1 (*p* = 0.004, *n* = 3 batches of cultures; Figures 3A,B) and WNK sh2 (*p* = 0.0025, *n* = 3 batches of cultures; Figures 3A,B) expressing neurons. No statistical difference was observed in the expression levels of NKCC1 or KCC2 (control sh vs. WNK3 sh1: NKCC1, KCC2, *p* > 0.9999; control sh vs. WNK3 sh2: NKCC1, *p* = 0.9121, KCC2, *p* = 0.5934; *n* = 3 batches of cultures; Figures 3C,D) between control and WNK3 knockdown neurons, although there seems to be a slight decreasing trend in the expression of NKCC1 and an increasing trend in the expression of KCC2 in WNK3 knockdown neurons (Figure 3A). Nonetheless, the phosphorylation of KCC2 at Thr1007, which is inversely correlated with the activity of KCC2 (Rinehart et al., 2009), was reduced in the membrane fractions of WNK3 sh1 and sh2 neurons as compared to control neurons (control sh vs. WNK3 sh1, *p* = 0.0233, *n* = 3 batches of cultures; control sh vs. WNK3 sh2, *P* = 0.0407, *n* = 3 batches of cultures; Figures 3E,G). On the other hand, the phosphorylation level of NKCC1 in WNK3 sh1 or sh2 expressing cells showed no significant statistical difference when compared to control neurons (Figures 3E,F). These data suggest that knockdown of WNK3 upregulates the activity of KCC2 but not NKCC1, which may be involved in the change in *E_GABA_* shift in the shWNK3 neurons.

To investigate if the hyperpolarizing shift of *E_GABA_* in mature WNK3-deficient neurons is due to an enhanced KCC2 activity, we employed a pharmacological approach in inhibiting KCC2 using a selective KCC2 inhibitor, VU0245501 (40 μM; Figures 3H,I). KCC2 inhibition significantly depolarizes* E_GABA_* (*F*_(1, 10)_= 23.18, *p* = 0.0007; Figure 3I), especially in WNK3 sh2 neurons (*P* = 0.0101, *n* = 5 neurons, baseline: −86.08 ± 10.61 mV; VU0245501: −41.15 ± 7.954 mV). Interestingly, inhibition of KCC2 also abolished the hyperpolarizing shift of *E_GABA_* in WNK-deficient neurons compared to control neurons, as there was no difference between control, WNK3 sh1 and WNK3 sh2 neurons after VU0245501 application (VU0245501: control: −48.26 ± 9.63 mV, *n* = 5; WNK3 sh1: −50.78 ± 8.92mV, *n* = 3; WNK3 sh2: −41.15 ± 7.954mV, *n* = 5; Figure 3I). These data suggest that the hyperpolarized shift of *E_GABA_* in WNK3-deficient neurons was due to decreased KCC2 phosphorylation and upregulation of KCC2 activity that in turn led to an overactive chloride efflux. Our findings are in agreement with previous studies demonstrating KCC2 is the major contributor to Cl^−^ levels in mature neurons (Conway et al., 2017).

### Morphological Changes Upon WNK3 Knockdown in Cultured Hippocampal Neurons

**Figure 4:**
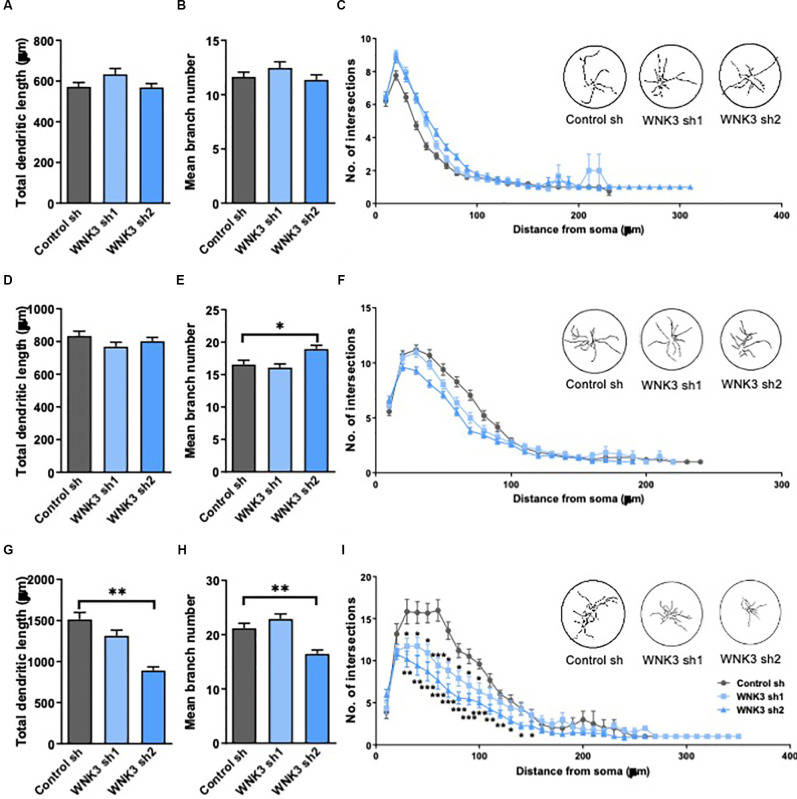
Morphologicalcharacterization of WNK3 knockdown in cultured hippocampal neurons.**(A,B)** Quantification of total dendritic length **(A)**and the number of dendritic branches **(B)** of control sh,WNK3 sh1, and sh2 neurons at DIV3, *n* = 55, 46, 55 neuronsfrom three batches of cultures. **(C)** Sholl plot comparing thedendritic arbors of control sh, WNK3 sh1, and sh2 neurons at DIV3.The x-axis represents the distance from the cell body, whilethe y-axis represents the number of intersections of a tracingwith a particular Sholl shell. The insets show representativetracings of neurons superimposed on circles denoting Sholl shells,*n* = 50 neurons in each group. **(D–F)** Quantification of total dendritic length **(D)**, number of dendritic branches **(E)** (*n* = 51, 57, 53 neurons from three batches of cultures), and the number of intersections **(F)** of control sh, WNK3 sh1 and sh2 neurons (*n* = 50 neurons in each group) at DIV7. **(G,H)** Quantification of total dendritic length **(G)** number of dendritic branches **(H)** of control sh, WNK3 sh1 and sh2 neurons at DIV14, *n* = 40, 48, 43 neurons from three batches of cultures. *p < 0.05 compared to control sh. One-way ANOVA with Bonferroni *post-hoc*. **(I)** the number of intersections of control sh, WNK3 sh1 and sh2 neurons at DIV14 (*n* = 10, 13, 15 neurons). Asterisk denotes comparison between control sh and WNK3 sh1, *n* = 10, 13 neurons; number sign denotes comparison between control sh and WNK3 sh2, *n* = 13, 15 neurons, *p < 0.05, **p < 0.01 and ***p < 0.001. One-way ANOVA with Bonferroni *post-hoc*.

In view of the established role of WNK3 and KCC2 in the maturation of neurons, and the hyperpolarized shift of *E_GABA_* and upregulated activity of KCC2 in WNK3 knockdown neurons, we next examined the morphological consequences of knocking down WNK3. Depolarizing GABAergic signaling has been reported to play a role in the morphological maturation of cells during early development (Cancedda et al., 2007; Salmon et al., 2020). Thus, we further explored the involvement of WNK3 in dendritic development of cultured neurons by measuring dendritic length, branching and complexity in immature (DIV3 and 7) and mature (DIV14) neurons. There were no differences between the control and shWNK neurons at DIV3 and DIV7 in total dendritic length (*F*_(2, 153)_= 2.339, *p* = 0.0942, one-way ANOVA followed by Dunnett’s test, control sh vs. WNK3 sh1, *p* = 0.1142, control sh vs. WNK3 sh2, *p* = 0.9916; control: *n* = 55 neurons, WNK3 sh1: *n* = 46 neurons, WNK3 sh2: *n* = 55 neurons; Figure 4A) and branch number (*F*_(2, 153)_= 1.317, *p* = 0.2710, control sh vs. WNK3 sh1, *p* = 0.3936, control sh vs. WNK3 sh2, *p* = 0.8856; control: *n* = 55 neurons, WNK3 sh1: *n* = 46 neurons, WNK3 sh2: *n* = 55 neurons; Figure 4B). Similarly, no difference at DIV7 in total dendritic length (*F*_(2, 158)_= 1.532, *p* = 0.2192, control sh vs. WNK3 sh1, *p* = 0.1445, control sh vs. WNK3 sh2, *p* = 0.5959; control: *n* = 51 neurons, WNK3 sh1: *n* = 57 neurons, WNK3 sh2: *n* = 53 neurons; Figure 4D), and branch numbers were observed across all groups, except for a slightly higher branch number in Wnk3 sh2 cells (*F*_(2, 164)_= 6.495, *p* = 0.0019, control sh vs. WNK3, *p* = 0.8091, control sh vs. WNK3 sh2, *p* = 0.0133; control: *n* = 51 neurons, WNK3 sh1: *n* = 57 neurons, WNK3 sh2: *n* = 59 neurons; Figure 4E). Notably, this higher branch number is not statistically significant when compared between different batches.

At DIV14, WNK3 knockdown neurons showed decreased total dendritic length when compared to control neurons (*F*_(2, 128)_= 20.99, *p* < 0.0001, control sh vs. WNK3 sh1, *p* = 0.0716, control sh vs. WNK3 sh2, *p* < 0.0001, control: *n* = 40 neurons, WNK3 sh1: *n* = 48 neurons, WNK3 sh2: *n* = 43 neurons; Figure 4G), and branch numbers (*F*_(2, 128)_= 14.65, *p* < 0.0001, control sh vs. WNK3 sh1, *p* = 0.2902, control sh vs. WNK3 sh2, *p* = 0.0006, control: *n* = 40 neurons, WNK3 sh1: *n* = 48 neurons, WNK3 sh2: *n* = 43 neurons; Figure 4H). Sholl analysis of neurite tracings that measures the number of intersections over increasing distance from the soma demonstrated a lesser dendritic complexity in WNK3 knocked down neurons at DIV14 (i.e., Distance from soma = 60 μm: control sh vs. WNK3 sh1, *p* = 0.0004, *n* = 10, 13 neurons; control sh vs. WNK3 sh2, *p* < 0.0001, *n* = 10, 15 neurons; Figure 4I), while no changes in dendritic complexity was observed in immature (DIV3 or DIV7) neurons (*n* = 50 in each group; Figures 4C,F). These results suggest that WNK3 knockdown has an influence on dendritic morphology in mature but not immature neurons.

### Electrophysiological Characteristics of WNK3 Knockdown in Cultured Hippocampal Neurons

The role of KCC2 affecting the excitatory synaptic function in mature neurons has been reported (Goutierre et al., 2019).

**Figure 5:**
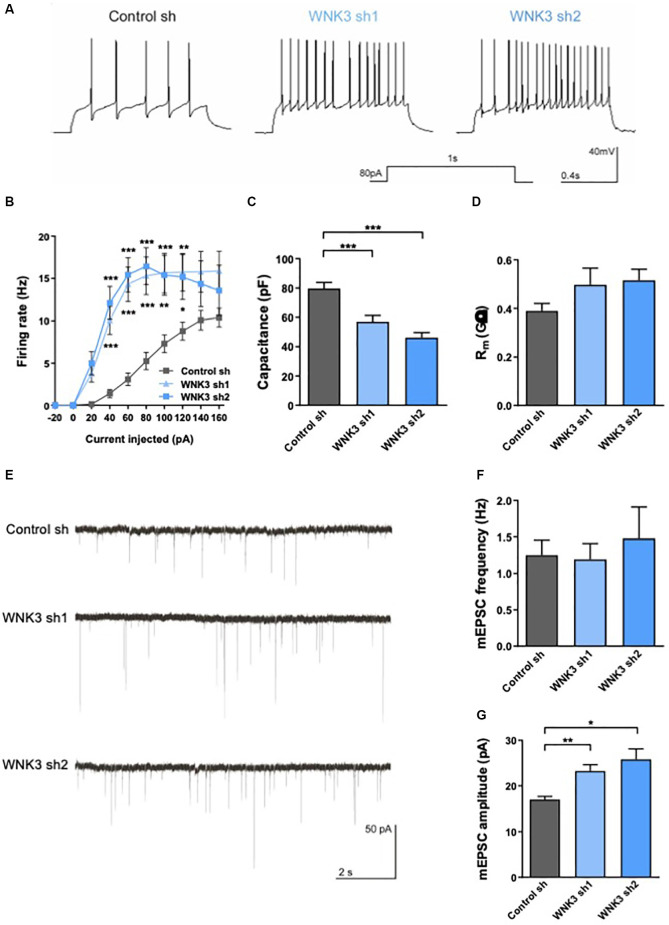
Electrophysiologicalcharacterization of WNK3 knockdown in cultured hippocampal neurons**(A)** action potentials (AP) recorded in control sh,WNK3 sh1 and sh2 neurons. **(B)** Input-output curves generated by plotting AP firing frequency response to current injection control sh: *n* = 38 neurons, WNK3 sh1: *n* = 28 neurons and WNK3 sh2: *n* = 25 neurons. *p < 0.05, **p < 0.01, ***p < 0.001 compared to control sh. Repeated-measure ANOVA with Bonferroni *post-hoc*. **(C,D)** Summary graphs of membrane properties of control sh (*n* = 38 neurons), WNK3 sh1 (*n* = 28 neurons) and WNK3 sh2 (*n* = 25 neurons) recorded in whole-cell patch clamp, including membrane capacitance **(C)** and membrane resistance **(D)**, ***p < 0.001 compared to control sh, One-way ANOVA with Bonferroni *post-hoc*. **(E)** Representative miniature excitatory postsynaptic current (mEPSC) recordings from control sh (*n* = 26 neurons), WNK3 sh1 (*n* = 19 neurons) and WNK3 sh2 (*n* = 5 neurons) DIV 14 hippocampal. **(F,G)** Graphs show mean mEPSC frequency **(F)** and amplitude **(G)**. **p < 0.01 and ***p < 0.001 compared to control sh. One-way ANOVA with Bonferroni *post-hoc*.

Previous work showed that KCC2 knockdown in hippocampal neurons led to reduced synaptic strength at glutamatergic inputs (Gauvain et al., 2011). By recording action potentials, we then characterized general electrophysiological characteristics and excitability of WNK3-deficient neurons (Figures 5A,B). Using an incremental current injection protocol, we recorded the input-output curves generated by plotting action potential (AP) firing frequency.

WNK3-deficient neurons generated AP more readily (Figure 5A) and exhibited higher firing rate in response to different current injection (*F*_(2, 88)_ = 12.74, *p* < 0.0001, repeat measure ANOVA, control sh: *n* = 38, WNK3 sh1: *n* = 28, WNK3 sh2: *n* = 25; i.e., control sh: 5.263 Hz ± 1.02 Hz, WNK3 sh1: 15.32 Hz ± 2.225 Hz, WNK3 sh2: 16.44 Hz ± 2.189 Hz, 80 pA current injection; Figure 5B). We also compared membrane properties, including membrane capacitance (Cm) and membrane resistance (Rm), that are correlated to the morphology of neurons (Liu et al., 1996). WNK3-deficient neurons have lower Cm than control neurons (*F*_(2, 88)_ = 19.42, *p* < 0.0001; control sh: 79.78 pF ± 3.937 pF, vs. WNK3 sh1: 57.14 pF ± 4.169 pF, *p* = 0.0002, *n* = 38, 28; control sh vs. WNK3 sh2: 46.2 pF ± 3.513 pF, *p* < 0.0001, *n* = 38, 25; Figure 5C), suggesting that WNK3-deficient neurons are smaller and less elaborated than control neurons. However, WNK3 deficiency has no significant effect on membrane resistance (*F*_(2, 86)_ = 2.234, *p* = 0.1133; control sh: 0.389.7 GΩ ± 0.0312 GΩ, vs. WNK3 sh1: 0.496 GΩ ± 0.0687 GΩ, *p* = 0.1914, *n* = 38, 26; control sh vs. WNK3 sh2: 0.5151 GΩ ± 0.0461 GΩ, *p* = 0.1153, *n* = 38, 25; Figure 5D). We next measured miniature excitatory postsynaptic currents (mEPSCs) by adding TTX and BMI to block voltage-gated sodium and GABA_A_R channels respectively (Figure 5E). WNK3-deficient neurons showed significant increase in mEPSC amplitude (*F*_(2, 16.25)_= 11.57, *p* = 0.0008, Brown-Frosythe ANOVA test; control: 17.06 ± 3.393 pA, vs. WNK3 sh1: 23.27 ± 5.874 pA, *p* = 0.0002, *n* = 26, 19; control sh vs. WNK3 sh2: 25.85 ± 2.263 pA, *p* = 0.0008, *n* = 26, 5; Figure 5G), indicating an upregulated postsynaptic function. No significant change was observed in the mean frequency (*F*_(2, 18.46)_= 0.1710, *p* = 0.8441, Brown-Frosythe ANOVA test; control: 1.249 ± 0.3152 Hz, vs. WNK3 sh1: 1.191 ± 0.3074, *p* = 0.9767, *n* = 26, 19; control sh vs. WNK3 sh2: 1.48 ± 0.4307, *p* = 0.8669, *n* = 26, 5; Figure 5F) from shWNK neurons when compared to control cells, suggesting the mean number of functional synapses was unaffected.

## Discussion

We found downregulated expression of WNK3 as cultured hippocampal neurons (*in vitro*) or animals (*in vivo*) matured, accompanied by an increase in KCC2 expression. Reduced WNK3 expression in cultured mature hippocampal neurons resulted in hyperpolarizing switch of *E_GABA_* through altered phosphorylation of KCC2 and decreased dendritic length and complexity, but enhanced neuron intrinsic excitability and synaptic excitation.

### KCC2 but Not NKCC1 Is Involved in WNK3’s Regulation of *E_GABA_* in Mature Neurons

The value of *E_GABA_*, representing the polarizing state of GABAergic signaling, is controlled by both NKCC1 and KCC2 during the development and maturation of neurons (Lu et al., 1999; Ben-Ari et al., 2012). In cortical and hippocampal neurons, CCCs control the equilibrium potential (*E_GABA_*) of GABA_A_R-mediated current and consequently regulate the efficacy of GABAergic inhibition (Yang et al., 2015). NKCC1 and KCC2 are two important kinases in GABAergic signaling under both normal and pathophysiological conditions, such as epilepsy (Zhu et al., 2008). Under certain conditions, changes in expressions and functions of KCC2 and/or NKCC1 regulate [Cl^−^] to affect the inhibitory influence of GABA (i.e., produce an excitatory or inhibitory shift; Rivera et al., 1999; Kim et al., 2011; Ben-Ari et al., 2012; Lee et al., 2015). We examined the expression of NKCC1 and KCC2 in hippocampal neurons *in vivo* and *in vitro*. Consistent with the results from *in vivo* experiments, expression of KCC2 in cultured neurons increased while WNK3 decreased as neurons mature. The phosphorylation of KCC2 at Thr1007 whose dephosphorylation is correlated with increased KCC2 activity (Rinehart et al., 2009), was downregulated significantly in WNK3-deficient neurons. However, neither NKCC1 expression nor its phosphorylation were affected over time in cultured neurons upon knocking down WNK3. These results suggest that WNK3 plays a part in regulating the shift of *E_GABA_*, likely through enhancing the phosphorylation of KCC2, but not NKCC1.

### WNK3 Mediated Differential Regulation of *E_GABA_* in Immature and Mature Neurons Is due to Different Expression Levels of KCC2 and NKCC1

WNK3 deficiency induced hyperpolarizing shift of *E_GABA_* in mature neurons, but not in immature neurons (Figures 2F–K). *E_GABA_* is mainly determined by the ([Cl^−^]i) in neurons (Jedlicka and Backus, 2006; Yang et al., 2010), which is controlled by the activity of KCC2 and NKCC1 cotransporters. In immature neurons, the activity of KCC2 is low, such that NKCC1-mediated Cl^−^ loading predominates, resulting in relatively high [Cl^−^]i. In mature neurons, KCC2-mediated Cl^−^ extrusion predominates leading to low [Cl^−^]i (Friedel et al., 2015). KCC2 phosphorylation at Thr^1007^, which has an inverse correlation with the activity of KCC2 (Rinehart et al., 2009), was downregulated in mature WNK3-deficient neurons (Figures 3A–E). However, there were no differences in the phosphorylation of NKCC1 in mature WNK3-deficient neurons when compared to control neurons. These results indicated that WNK3 knockdown upregulated the activity of KCC2 but not NKCC1 in mature neurons. Due to WNK3 knockdown-induced upregulation of KCC2, the efflux of Cl^−^ is enhanced, leading to *E_GABA_* hyperpolarization. But in immature WNK3-deficient neurons, the mechanism underlying unchanged *E_GABA_* is not clear. Friedel et al. (2015) found that the reduction of WNK1 caused a ~15 mV hyperpolarizing shift of E_GABA_through upregulating KCC2 activity in immature neurons but not mature ones. Therefore, it is likely that the regulations of WNK1 or WNK3 on KCC2 are different in immature and mature neurons. It is possible that there are other regulatory factors contributing to regulating KCC2 in immature neurons in the developing brain.

### WNK3 Deficiency Induced Morphological Alterations Associated With Changes of GABA Inhibition Regulated by KCC2

WNK3 knockdown had no influence on the dendritic morphology of immature neurons, but mature WNK3-deficient neurons showed decreased total dendritic length and complexity, and slight alteration in branch numbers as compared to controls (Figures 4A–I). Excitatory GABA was previously shown to be involved in the morphological maturation of adult newly generated hippocampal granule cells *in vivo* (Deisseroth et al., 2004; Tozuka et al., 2005; Ge et al., 2006). Specifically, we have previously found that conversion of GABAergic excitation into inhibition by reducing the expression of NKCC1, induced significant decreases in total dendritic length and branch number, as well as dendritic complexity of new neurons in the adult hippocampus (Ge et al., 2006). Similarly, premature up-regulation of KCC2 in the embryo leading to an early termination of GABA excitation *in vivo* significantly dampened dendritic growth and branching of pyramidal neurons in the neonatal cortex at P14 (Cancedda et al., 2007). Thus, GABA excitation appears to be critical for neuronal morphogenesis. Moreover, overexpression of a mutated form of KCC2 (mtKCC2) defective in Cl^−^ transporter activity, showed no morphological impairment in neurons (Cancedda et al., 2007), indicating that the impaired morphological maturation by premature KCC2 expression could be attributable to the altered transporter function of KCC2 that eliminated the GABA excitation. In our study, WNK3 deficiency enhanced the transporter function of KCC2 that promoted GABA inhibition in mature neurons. Our findings showing the upregulation of KCC2 and morphological defects in mature WNK3-deficient neurons are consistent with morphological changes reported in other studies manipulating expression and/or activation of KCC2 or NKCC1 (Ge et al., 2006; Cancedda et al., 2007). However, the exact mechanism by which WNK3 regulates neuronal maturation is still not clear, and there may be other factors involved. Given the predominant role of NKCC1 in immature neurons, we did not observe significant expression changes in NKCC1 upon WNK3 knockdown, and morphological changes were not detected in immature WNK3-deficient neurons at DIV 4 or 7.

### WNK3 Knockdown Induced an Increase in Intrinsic Neuronal Excitability

Electrophysiological properties measured from the mature shWNK3 neurons indicated an upregulation of intrinsic excitability with a higher firing rate and decreased membrane capacitance compared to controls. Membrane capacitance value reflects cell surface area (Gilbertson, 2002) and is a fundamental neuronal property. Thus, measurements of cellular capacitance have been used to normalize for variability in cell size of different neurons (Turrigiano et al., 1995; Schulz et al., 2006; Khorkova and Golowasch, 2007). The decrease in capacitance in WNK3-deficient neurons indicates smaller cell surface area, likely due to less elaborated arborization and/or smaller size. Studies on various cell types have shown that CCCs can exert a major influence on cell morphology, such as volume, *via* actions mediated by changes in the intracellular chloride concentration ([Cl^−^]i; Russell, 2000; Adragna et al., 2004). As a transporter of Cl^−^, KCC2 also regulates the cell volume in response to osmotic stress and/or changes in [Cl^−^]i (Lang et al., 1998; Kahle et al., 2005).

The firing rate of AP in mature WNK3-deficient neurons is obviously enhanced, indicating an increase in neuronal intrinsic excitability (Morton et al., 2015; Chung and Bailey, 2018). A recent study reported chronic KCC2 knockdown in adult rat dentate granule cells that resulted in little effect on GABA signaling at rest, but increased neuronal excitability (Goutierre et al., 2019). This conditional chronic KCC2 ablation showed a ~20% decrease in mEPSC amplitude but no change in frequency, consistent with our data showing a 25% increase in mEPSC amplitude and no change in frequency in WNK3-deficient neurons where KCC2 activity was significantly enhanced. These KCC2 ablation-induced enhanced excitability and EPSP/spike coupling are suggested to be likely due to the reduced expression of Task-3 potassium channels, independent of GABA signaling (Goutierre et al., 2019). Interestingly, hippocampal and cerebellar granule cells, as well as CA1 pyramidal cells strongly express Task-3 channels (Ramadoss et al., 2008; Marinc et al., 2014), whereas Purkinje cells predominantly express Task-1 and Task-5 (Karschin et al., 2001; Ramadoss et al., 2008). Thus, the differential expression of potassium channels in different cell types may underlie the difference between our results on the effects of KCC2 on neuron excitability. Although the connection between WNK3 and neuronal excitation is generally thought to be *via* modulation of chloride transporters, it is possible that other KCC2-independent mechanisms are involved in modulating neuron excitability in WNK3 knockdown neurons. For example, Fox-1 has been shown to alter transcripts encoding amounts of genes involved in autism spectrum disorder and is responsible for generating proper alternative splicing variants required for normal neuronal excitability and synaptic transmission (Voineagu et al., 2011; Lee et al., 2012). A previous study had revealed a corresponding increase in neuronal excitability in the dentate gyrus of *Rbfox1* (RNA-binding Fox) knockout mice (Gehman et al., 2011). Lee et al. (2012) reported a relationship between WNK3 and Rbfox1 and found that WNK3 could bind to splicing regulator Rbfox1 and suppress its splicing activity through a kinase activity-dependent cytoplasmic relocalization of Rbfox1 (Lee et al., 2012). We also observed reduced levels of Fox splicing factors in WNK3 knockdown neurons (data not shown). Thus, we speculate that WNK3 deficiency-induced upregulation of neuron excitability in mature neurons may be due to its effect on splicing regulator Rbfox1. The exact mechanism underlying WNK3’s effects on neuronal excitability remains to be further explored.

### WNK3 Deficiency Increased Synaptic Excitation Is Affected by Postsynaptic AMPAR Under the Control of KCC2

We demonstrated a significant increase in the amplitude but no change in the frequency of mEPSCs in mature WNK3-deficient neurons, consistent with a previous report showing that suppression of KCC2 expression decreased the amplitude of mEPSC with no effect on the frequency (Gauvain et al., 2011). These data suggest that the alteration of mEPSC induced by KCC2 suppression may reflect a reduced density of postsynaptic AMPA receptors (AMPAR) at excitatory synapses. The C-terminal domain (CTD) of KCC2 interacts with 4.1 N protein (Li et al., 2007), which binds both actin and GluR1 subunit of AMPAR (Shen et al., 2000). Direct KCC2 interaction with actin-associated proteins (such as 4.1 N) contributes to forming a molecular barrier hindering the lateral diffusion of transmembrane proteins (such as AMPAR) within dendritic spines (Gauvain et al., 2011). Disrupting this barrier by KCC2 suppression using RNAi or preventing its interaction with submembrane proteins by overexpressing KCC2-CTD promotes GluR1 lateral diffusion and disperses the receptor pools and then the synaptic pools, leading to decreased efficacy of excitatory synapses. However, this change was not observed upon treatment with the KCC2-specific antagonist VU0240551 (Gauvain et al., 2011). Furthermore, alteration in diffusion and clustering of KCC2 near excitatory synapses may further affect the synaptic plasticity of neurons (Chevy et al., 2015). Thus, membrane expression but not transport function of KCC2 is likely to be involved in controlling the decrease of postsynaptic AMPAR aggregation.

Surface expression and trafficking of KCC2 are thought to be crucially regulated by its (de)phosphorylated state (Roussa et al., 2016). The PKC pathway-dependent phosphorylation of KCC2 at S940 increases its cell surface expression and promotes KCC2 membrane stability in cultured hippocampal neurons (Lee et al., 2007). In contrast, Src-mediated Y903 and Y1087 phosphorylation decrease the cell surface stability of KCC2 by enhancing its lysosomal degradation (Lee H. H. et al., 2010). While WNKs-dependent (de)phosphorylation at T906 and T1007 affect KCC2 transport function (Rinehart et al., 2009), its role in membrane trafficking has not been demonstrated. Besides (de)phosphorylation, other molecular pathways that influence the membrane expression of KCC2 in immature neurons include TrkB and 5-HT2A serotonin receptors (Khirug et al., 2005; Bos et al., 2013). However, for mature neurons, the other molecular mechanisms regulating KCC2 membrane trafficking remains to be elucidated.

Thus, enhanced synaptic excitation in mature WNK3-deficient neurons in our study is most possibly due to the alteration of membrane expression of KCC2, leading to a change of lateral diffusion of AMPAR. However, the involvement of phosphorylation or other pathways on KCC2 membrane trafficking warrants further investigations.

To our knowledge, this study provides the first evidence that WNK3 plays a crucial role in maintaining the polarity of GABAergic signaling, neuron morphology, intrinsic excitability, and synaptic excitation in mature hippocampal neurons in either a KCC2-dependent or independent manner. Our findings demonstrate that WNK3 deficiency induces *E_GABA_* hyperpolarization, indicating an increase in GABA inhibitory response in mature neurons, through the upregulation of KCC2 activity. In addition, WNK3 deficiency in mature neurons leads to alterations in neuron morphology consistent with the shift of GABAergic inhibitory response upon upregulation of KCC2 activity. Other electrophysiological characteristics, including enhancement of intrinsic excitability and synaptic excitation, were also affected in WNK3-deficient neurons. WNK3 possibly affects neuronal somatic and synaptic properties by modulating KCC2 activity, resulting in abnormal activity patterns that may underlie psychiatric and neurological disorders. However, much remains to be explored to better define the mechanisms underlying WNK3’s regulation of KCC2 activity and downstream pathways that may have wide implications in biological regulations.

### Data Availability Statement

The original contributions presented in the study are included in the article, further inquiries can be directed to the corresponding author/s.

### Ethics Statement

The animal study was reviewed and approved by IACUC Singhealth, Singapore (Protocols 2014/SHS/698 and 2018/SHS/1155) for dissection and isolation of primary rat neurons.

## Author Contributions

WL designed and performed all electrophysiology recordings and analysis, western blot analyses, prepared figures, and drafted the manuscript. EC carried out *in vitro* morphological experiments and analysis. TC analyzed some data and wrote the manuscript. BT provided critical input for the project. EG conceived the project, designed the experiments, and wrote the manuscript.

## Conflict of Interest

The authors declare that the research was conducted in the absence of any commercial or financial relationships that could be construed as a potential conflict of interest.

## Publisher’s Note

All claims expressed in this article are solely those of the authors and do not necessarily represent those of their affiliated organizations, or those of the publisher, the editors and the reviewers. Any product that may be evaluated in this article, or claim that may be made by its manufacturer, is not guaranteed or endorsed by the publisher.
